# Comparison of ddRADseq and EUChip60K SNP genotyping systems for population genetics and genomic selection in *Eucalyptus dunnii* (Maiden)

**DOI:** 10.3389/fgene.2024.1361418

**Published:** 2024-03-26

**Authors:** Natalia Cristina Aguirre, Pamela Victoria Villalba, Martín Nahuel García, Carla Valeria Filippi, Juan Gabriel Rivas, María Carolina Martínez, Cintia Vanesa Acuña, Augusto J. López, Juan Adolfo López, Pablo Pathauer, Dino Palazzini, Leonel Harrand, Javier Oberschelp, Martín Alberto Marcó, Esteban Felipe Cisneros, Rocío Carreras, Ana Maria Martins Alves, José Carlos Rodrigues, H. Esteban Hopp, Dario Grattapaglia, Eduardo Pablo Cappa, Norma Beatriz Paniego, Susana Noemí Marcucci Poltri

**Affiliations:** ^1^ Instituto de Agrobiotecnología y Biología Molecular, UEDD INTA-CONICET, Hurlingham, Argentina; ^2^ Laboratorio de Bioquímica, Departamento de Biología Vegetal, Facultad de Agronomía, Universidad de la República, Montevideo, Uruguay; ^3^ Estación Experimental Agropecuaria de Bella Vista, Instituto Nacional de Tecnología Agropecuaria, Bella Vista, Argentina; ^4^ Instituto de Recursos Biológicos, Instituto Nacional de Tecnología Agropecuaria, Hurlingham, Argentina; ^5^ Estación Experimental Agropecuaria de Concordia, Instituto Nacional de Tecnología Agropecuaria, Concordia, Argentina; ^6^ Facultad de Ciencias Forestales, Universidad Nacional de Santiago del Estero (UNSE), Santiago del Estero, Argentina; ^7^ Centro de Estudos Florestais e Laboratório Associado TERRA, Instituto Superior de Agronomia, Universidade de Lisboa, Tapada da Ajuda, Lisboa, Portugal; ^8^ Empresa Brasileira de Pesquisa Agropecuária (EMBRAPA), Recursos Genéticos e Biotecnologia, Brasilia, Brazil; ^9^ Consejo Nacional de Investigaciones Científicas y Técnicas, Buenos Aires, Argentina

**Keywords:** double digest restriction-site associated DNA sequencing, genotyping by sequencing, SNP array, genomic prediction, ABLUP, GBLUP

## Abstract

*Eucalyptus dunnii* is one of the most important *Eucalyptus* species for short-fiber pulp production in regions where other species of the genus are affected by poor soil and climatic conditions. In this context, *E. dunnii* holds promise as a resource to address and adapt to the challenges of climate change. Despite its rapid growth and favorable wood properties for solid wood products, the advancement of its improvement remains in its early stages. In this work, we evaluated the performance of two single nucleotide polymorphism, (SNP), genotyping methods for population genetics analysis and Genomic Selection in *E. dunnii*. Double digest restriction-site associated DNA sequencing (ddRADseq) was compared with the EUChip60K array in 308 individuals from a provenance-progeny trial. The compared SNP set included 8,011 and 19,008 informative SNPs distributed along the 11 chromosomes, respectively. Although the two datasets differed in the percentage of missing data, genome coverage, minor allele frequency and estimated genetic diversity parameters, they revealed a similar genetic structure, showing two subpopulations with little differentiation between them, and low linkage disequilibrium. GS analyses were performed for eleven traits using Genomic Best Linear Unbiased Prediction (GBLUP) and a conventional pedigree-based model (ABLUP). Regardless of the SNP dataset, the predictive ability (PA) of GBLUP was better than that of ABLUP for six traits (Cellulose content, Total and Ethanolic extractives, Total and Klason lignin content and Syringyl and Guaiacyl lignin monomer ratio). When contrasting the SNP datasets used to estimate PAs, the GBLUP-EUChip60K model gave higher and significant PA values for six traits, meanwhile, the values estimated using ddRADseq gave higher values for three other traits. The PAs correlated positively with narrow sense heritabilities, with the highest correlations shown by the ABLUP and GBLUP-EUChip60K. The two genotyping methods, ddRADseq and EUChip60K, are generally comparable for population genetics and genomic prediction, demonstrating the utility of the former when subjected to rigorous SNP filtering. The results of this study provide a basis for future whole-genome studies using ddRADseq in non-model forest species for which SNP arrays have not yet been developed.

## 1 Introduction

The genus *Eucalyptus* comprises more than 700 species of native trees from Australia, New Guinea, Timor, Indonesia and the Philippines (Ladiges et al., 2003; Thornhill et al., 2019), with some of them exhibiting excellent growth and adaptability in different environments. The most widely planted species include *E. grandis*, *E. saligna*, *E. pellita*, *E. urophylla*, *E. globulus*, *E. dunnii*, *E. nitens*, *E. tereticornis* and *E. camaldulensis* (Grattapaglia and Kirst, 2008). Despite its limited natural distribution in Australia, *E. dunnii* (Maiden, 1905) has shown relevant genetic variability for growth, adaptation to different soils and tolerance to abiotic stresses such as frost, drought and high summer humidity, when planted as an exotic (Darrow, 1994; Jovanovic et al., 2000; Clarke, 2009; Thomas et al., 2009; Shi et al., 2016). As a result of these advantages, *E. dunnii* plantations have been established in subtropical areas such as southern China, South Africa, South America and Australia, where it is considered to be an alternative to other species, particularly in the context of climate change (Marcó and White, 2002; Marcó, 2005; Maseko et al., 2007; Hardner et al., 2016; López et al., 2016; Shi et al., 2016; Gallo et al., 2018; Resquin et al., 2019). However, genetic improvement of *E. dunnii* is still at an early stage.

Molecular breeding programs facilitate the selection of the best individuals in the early stages of development, before the phenotypic traits of interest have been expressed. It therefore has significant potential to accelerate the rate of genetic gain in a shorter time, which is extremely important in forest species with long generation times (Neale and Kremer, 2011; Varshney et al., 2017). Numerous studies have been conducted in forest trees demonstrating that Genomic Selection (GS) equals or outperforms phenotypic selection for traits related to growth and wood properties. This enhances the rate of genetic improvement over time by intensifying selection, significantly reducing generation intervals, and improving the precision of breeding values (Grattapaglia, 2022).

In GS, the underlying assumption is that all markers might be tied to genomic loci influencing the trait under study and therefore can be used to select the best individuals for breeding (Meuwissen et al., 2001) through the application of novel statistical methods based on whole genome regression (Cappa et al., 2019). Unlike marker-assisted selection (MAS), which applies stringent hypothesis testing to declare the association of markers with variation in the target trait, GS relies on capturing all loci that cause phenotypic variation among individuals with dense marker coverage (Hayes and Goddard, 2010; Isik, 2014).

By building prediction models based on the analysis of a population with both phenotypes and genotypes data (Training population, TP), the sum of marker effects can be used to predict the genomic estimated breeding values (GEBVs) of individuals that were only genotyped (Selection candidates, SC). It is a consensus now that GS depends largely on the existence of genetic relatedness between the TP and SC and some degree of Linkage Disequilibrium (LD) between markers and causal loci (Desta and Ortiz, 2014; Tan et al., 2017).

A commonly used GS approach is the or Genomic Best Linear Unbiased Prediction (GBLUP, Strandén and Garrick, 2009; VanRaden, 2008). This method predicts breeding values using a genomic-based relationship matrix between individuals (***G***-matrix). By using the ***G***-matrix instead of the conventional matrix of expected pedigree relationships (***A***-matrix), GBLUP predicts the GEBVs more accurately, thus increasing the genetic gain and selection accuracy for the next-generation of the breeding cycle (Nejati-Javaremi et al., 1997; Villanueva et al., 2005).

In recent decades, different types of molecular markers have been developed for genetic analysis, such as Single Nucleotide Polymorphism (SNP), Simple Sequence Repeats (SSR) or Microsatellites, Insertions/Deletions (InDels), Structural Variants (SV), etc (Fuentes-Pardo and Ruzzante, 2017). SNPs have become the markers of choice due to their abundance, stability, codominance, low cost per datapoint and ease in assay design, automation and data interpretation (Bajgain et al., 2016; Torkamaneh et al., 2018).

The ideal genotyping technique for GS would be Whole Genome Sequencing (WGS) providing the full sampling of genetic variants among individuals (Fuentes-Pardo and Ruzzante, 2017). Nevertheless, this is still too expensive to accommodate experiments with large sample sizes, especially for forest tree breeding programs that operate on low budgets. SNP arrays and reduced representation sequencing (RRS) have been used in practice to obtain genome-wide SNP data in a cost-effective way for molecular breeding studies. Both methodologies have their advantages and disadvantages (Pavan et al., 2020).

For commercially relevant species of *Eucalyptus*, multispecies SNP genotyping platforms were developed including the Illumina Infinium based EUChip60K (Silva-Junior et al., 2015) and the second generation Euc72K array based on the Axiom technology^1^. These two systems allow genotyping over 60,000 genome-wide SNPs, have approximately 28,000 SNPs in common and satisfy essential requirements of high precision, throughput, data reproducibility and low genotyping cost (Silva-Junior et al., 2015). The SNPs included in these arrays were discovered from low-depth WGS data of 240 individuals of 12 different species and detected using a reference genome of *E. grandis* v1.0 (Myburg et al., 2014). *E. dunnii* was represented by only 12 unrelated individuals in which 17,014 SNPs were converted with Minor Allele Frequency (MAF) > 0.01 (Silva-Junior et al., 2015). Although these arrays have been very successful worldwide for they accommodate several different eucalypt species, SNP discovery from a small number of individuals is subject to ascertainment bias (Albrechtsen et al., 2010; Li and Kimmel, 2013).

Medium-density SNP arrays with several tens of thousands of SNP, have been developed for all mainstream forest tree species, such as for *Populus trichocarpa* (Geraldes et al., 2013), *Picea* ssp. (Pavy et al., 2013; Bernhardsson et al., 2021), *Pinus* ssp. (Plomion et al., 2015; Perry et al., 2020; Caballero et al., 2021; Graham et al., 2022; Jackson et al., 2022; Kastally et al., 2022), *Pseudotsuga menziesii* (Howe et al., 2020), *Araucaria angustifolia* (Silva et al., 2020), *Eucalyptus* ssp. (EUChip60K and Euc72K). In the case of *Eucalyptus*, the two SNP arrays have been used in a large number of studies (e.g., Telfer et al., 2015; Müller et al., 2019; Marco de Lima et al., 2019; Mhoswa et al., 2020; Ballesta et al., 2018; Mostert-O'Neill et al., 2020; Jurcic et al., 2021; Paludeto etal., 2021; Valenzuela et al., 2021; Tan and Ingvarsson, 2022; Duarte et al., 2023). Given its multispecies nature, this platform generally provides between 10,000 and 30,000 informative SNPs in all planted species. Due to its fixed content it is unclear whether population genomics analyses ultimately exclude relevant variants in unsampled genomic regions.

As a practical alternative to SNP array development, especially for orphan species, SNP genotyping based on RRS strategies have been used. SNP genotyping following Restriction Enzyme based RRS (REbRRS) techniques are approaches that combine genome reduction and sampling of both coding and non-coding regions without the need for prior genomic information (Davey et al., 2011; Andrews et al., 2016). These techniques rely on Next-Generation Sequences (NGS) of a reduced genome portion of several individuals analysed simultaneously (multiplexed), do not require a reference genome or prior knowledge of polymorphisms, and combine marker discovery and genotyping in a single protocol. Therefore, they provide a rapid, high-throughput, and cost-effective strategy for performing genome-wide analyses. Furthermore, they can be applied to non-model species and unique germplasm sets to obtain exclusive polymorphism information (Davey et al., 2011; Andrews et al., 2016), to sample alternative genomic regions providing complementary data to SNP array data, especially in plants (Deschamps et al., 2012).

REbRRS embraces a group of similar protocols that include Genotyping by Sequencing (GBS, Elshire et al., 2011), Restriction site Associated DNA sequencing (RADseq, Baird et al., 2008), and double digest RADseq (ddRADseq, Peterson et al., 2012), being widely adopted in the conservation and breeding area (Andrews et al., 2016; Fuentes-Pardo and Ruzzante, 2017; Campbell and Dupuis, 2018; Wright et al., 2019). In comparison to fixed content SNP arrays, RRS techniques require bioinformatics analysis of sequence data to detect variants and declare genotypes (Nielsen et al., 2011). Missing data, loci sampling and genotype reproducibility issues across experiments are common features of these methods due to polymorphisms in enzyme cleavage sites and variation in the sequence coverage across individuals and loci (Andrews et al., 2016), potentially causing bias in population genetic statistics. To mitigate these limitations, stringent filtering for high call rates and imputation are highly recommended (Money et al., 2015; Andrews et al., 2016; Bajgain et al., 2016; Torkamaneh et al., 2018). Both methods are powerful means to study the genome and provide sufficient resolution to perform different molecular genetic approaches, despite the fact that chip-based SNP genotyping requires less computational knowledge and data processing resources than the REbRRS method, resulting in less missing data and higher reproducibility (Bajgain et al., 2016).

REbRRS methods have been applied to forest species mainly for linkage mapping, QTL detection, marker development, phylogenetics, phylogeography, parentage analysis, association mapping, genomic selection, population genetics, genome scanning, among others (Parchman et al., 2018). In particular, ddRADseq combined with reference genomic SNP calling yielded a higher number of reliable markers compared to other REbRRS methods such as GBS and RADseq in beech and oak Ulaszewski et al. (2021). In *Eucalyptus*, a few studies have used REbRRS methods, including GBS (Grattapaglia et al., 2011; Durán et al., 2018; Klápště et al., 2021), DArT-seq (Sansaloni et al., 2011; Marco de Lima et al., 2019), and ddRADseq (Aguirre et al., 2019). Specifically, for *E. dunnii* ddRADseq has been optimized (Aguirre et al., 2019), and there is a scale-up protocol (Aguirre et al., 2023).

In this work, we were interested in evaluating the comparative performance of two high-throughput genotyping systems, ddRADseq and the SNP platform EUChip60K, for population genetics analyses and GS. To our knowledge, this is the first report where a restriction enzyme-based RRS method, ddRADseq, is compared to SNP array data for population genetic parameters and GS analyses in a tree species, and specifically in *Eucalyptus*.

## 2 Materials and methods

### 2.1 *Eucalyptus dunnii* breeding population

The *E. dunnii* breeding population (1,520 trees) under study was established in 1991 (31° 45′S, 58° 15′W, 40 m. a.s.l., Entre Ríos province, Argentina) with a complete block design (Marcó and White, 2002). This population was composed of 72 open-pollinated (OP) families, of which 60 were from four native origins in New South Wales (NSW) state in Australia. The remaining 12 families came from a local provenance or seed sources of known Australian origin (Moleton, NSW), where they were selected by their superiority in stem straightness and volume (Supplementary Table S1).

### 2.2 Phenotypic characterization of *E. dunnii* breeding population

*E. dunnii* trees were measured for growth traits by Diameter at Breast Height at six and 20 years old (DBH6, and DBH20) and Stem Straightness at 6 years old (SS6; López et al., 2016; Marcó and White, 2002). At 20 years old, the intensity of growth stresses was evaluated by measuring the Log End Split Index (LESI20, López et al., 2016), and Wood basic Density (WD20, Kg/m^3^) was estimated by water immersion. Estimates of six wood chemical properties at 20 years were obtained using Near Infrared spectroscopy (NIR) at the Instituto Superior de Agronomia (ISA, Portugal). These included: Cellulose content (CEL20), Total and Ethanolic extractives (TE20 and EE20), Total and Klason Lignin content (TL20 and KL20) and Syringyl and Guaiacyl lignin monomer ratio (S/G20), as described by Rodrigues et al. (1998). Details of the traits measured are summarized in Supplementary Table S2. Phenotypic traits data were adjusted to normal distributions and standardized if required, except for the SS6 categorical variable, which was transformed to a continuous variable using a Normal Score (*stats* R package, R Core Team, 2023). The experimental design effect was removed using breedR (Muñoz and Sánchez, 2014), with an individual tree mixed linear model using restricted maximum likelihood inference (REML, Patterson and Thompson, 1971).

### 2.3 Genotypic data of *E. dunnii* breeding population

DNA was extracted from lyophilized leaves of 308 *E. dunnii* individuals using a CTAB method (Hoisington et al., 1994) with modifications for the species (Marcucci Poltri et al., 2003), quantified with Qubit 2.0 fluorometer (Thermo Fisher Scientific), and quality verified by both Nanodrop (Thermo Fisher Scientific) and 1% agarose gel electrophoresis (as described in Aguirre et al., 2019; 2023).

#### 2.3.1 SNP ddRADseq dataset

A ddRADseq genotyping protocol optimized for *E. dunnii* (Protocol 2 from Aguirre et al., 2019; Aguirre et al., 2023) was applied to the breeding population, by constructing 13 libraries/pool of 24 samples each (including four extra samples, as required by the protocol), at the Unidad de Genómica, IABiMo-INTA, Argentina. The libraries were sequenced using a NextSeq 500 instrument (Illumina, Inc., San Diego, CA, USA). Sequencing was carried out with a 150-cycle high-output kit NextSeq and set up for 75 bp paired-end (PE) reads (Illumina Inc.).

To search for loci and SNP markers in the ddRADseq data, Stacks v1.48 software (Catchen et al., 2013) was used, as described by Aguirre et al. (2019) for “with reference analysis”. In summary, sequences were filtered by quality with *process_radtags* (removing barcodes, adapters, reads without enzyme cutting site, and with Phred quality value mean below 10, also truncating them to 66 bp). The loci and SNPs were identified using the *ref_map.pl* pipeline, where reads were previously mapped against the *E. grandis* reference genome v2.0 (Myburg et al., 2014) using Bowtie2 (Langmead and Salzberg, 2012) with default parameters. In detail, a minimum of three reads was used to define an allele (tag or stack) within an individual (-m 3), two bases of difference between alleles were allowed to build a locus within an individual (-M 2), and two different bases between loci were allowed to build the loci catalogue (-n 2) between individuals. Stacks or alleles with great depth of sequences were removed since it is very likely that they came from repetitive regions of the genome (-t). As a diploid species, only loci made up of two stacks (-X “ustacks: -max_locus_stacks 2″) were considered. Additionally, the *rxstacks* program was applied as described by Aguirre et al. (2019); however, in this case, the loci logarithm of the likelihoods was filtered up to −20 (minus 20). Finally, the *populations* component was executed using a filter of defined loci with a minimum of six reads (-m 6), as the call of heterozygous loci is more robust as the ddRADseq read depth increases (from 3 to 6; Rochette and Catchen, 2017).

#### 2.3.2 SNP EUChip60K dataset

DNA samples from 308 individuals were lyophilized in 96-well plates and sent to the NEOGEN (USA, ^©^ Neogen Corporation) for genotyping with the EUChip60K (Silva-Junior et al., 2015). For allelic designation, the genotyping module of GenomeStudio 2.0 program was used (Illumina, San Diego, CA, USA). A cluster file optimized for the Maidenaria section of subgenus *Symphyomyrtus* (i.e.,: *E. globulus*, *E. nitens* and *E. dunnii*) and a technical filter for quality parameters were used as suggested by Silva-Junior et al. (2015).

Because the SNP coordinates were provided based on the *E. grandis* reference genome v1, oligonucleotide sequences of chip probes were mapped against the *E. grandis* v2.0 reference genome using Bowtie2 (Langmead and Salzberg, 2012; with default parameters). Next, to compare with the ddRADseq dataset, the SNP coordinates of the EUChip60K dataset were converted to the *E. grandis* v2.0 genome using our own script in bash/R language.

### 2.4 Genomic datasets quality filter and imputation

The proportion of total and per genotype missing data, observed heterozygosity per individual, and genetic relationships between them were calculated using PLINK v1.9 (Chang et al., 2015). An individual was eliminated from both datasets using VCFtools software (Danecek et al., 2011) if it showed at least one of the following occurrences in at least one SNP dataset: a high proportion of missing data (more than 60%), high heterozygosity values (outside the range of population distribution, higher than three times the standard deviation), or unexpected genetic relationships with other individuals (greater than expected for an OP population, such as father or mother/child relationship and/or very close to clones, --king-cutoff 0.354). SNPs were then filtered out using a MAF of 0.01 by PLINK v1.9. As a final quality control of the filtered data, pairwise genetic distances were calculated with each genotyping dataset using the snpgdsIBS option of the SNPRelate R package (Zheng et al., 2012). Both datasets were correlated with a Mantel test (Mantel, 1967; vegan R package; Dixon, 2003).

Imputation of missing data, in both genotypic datasets, was performed using the LinkImpute program (Money et al., 2015). After imputation, ddRADseq and EUChip60K datasets were merged with the BCFtools tool (Li et al., 2009) to generate a third joint SNP dataset (hereinafter called ddRADseq + EUChip60K or combined data).

### 2.5 Population genetics analyses

#### 2.5.1 SNP distribution and MAF in the three datasets

To compare the performance of the applied genotyping methodologies and the combined data, SNP distributions and their allele frequencies along the *E. grandis* genome were evaluated by CMplot^2^ and synbreed R packages (Wimmer et al., 2012).

#### 2.5.2 Linkage disequilibrium estimation

The LD between each SNP pair was estimated using TASSEL software (Bradbury et al., 2007) considering SNPs with MAF ≥0.01 and no correction for population structure or relatedness as Estopa et al. (2023). Patterns of LD decay for each dataset were plotted in a 10 Kbp window using R software according to the method of Hill and Weir (1988) and based on the physical distance of the *E. grandis* v2 genome (Myburg et al., 2014).

#### 2.5.3 Population genetic structure and diversity parameter estimation

To estimate genetic structure and genetic diversity parameters, each dataset was LD pruned (*r* ^2^ greater than 0.2) by implementing the --indep-pairwise function of the PLINK v1.9 program, using 2 Mb windows with an overlap between them of 200 kb. This filter was used to eliminate redundant information, obtain a more accurate estimation, and reduce the computational demand of statistical analyses.

Population genetic structure was estimated by Discriminant Analysis of Principal Components (DAPC; Jombart et al., 2010) using adegenet 2.0.0 R package (Jombart, 2008). Because DAPC requires defining the number of groups in advance, SNP data was transformed using PCA and a k-means clustering algorithm. Successive k-means were run using find. clusters function of adegenet, and optimal grouping was chosen through the lowest Bayesian Information Criterion (BIC; Schwarz, 1978) value. For these population structure analyses, random sub-sampling of 800 SNPs was applied to each of the three genomic datasets filtered by LD. Subsequently, F_ST_ was calculated between the genetic groups defined by DAPC for each of the three datasets using the populations module (option: -fstats) of the Stacks program (Catchen et al., 2013). The significance of each F_ST_ value was calculated through bootstrap resampling implemented in the said population module (--fst_correction *p*_value -k --bootstrap_fst). This calculation was used with the default parameters, which were a resampling number of 10,000 times and a *p*-value less than 0.05, to report the F_ST_ value.

The following population genetic diversity statistics were calculated for each dataset and genetic structure group: allele frequencies p and q, expected (He) and observed (Ho) Heterozygosity and Polymorphic Information Content (PIC) with the popgen function in the snpReady R package (Granato et al., 2018).

### 2.6 Genomic selection

#### 2.6.1 Genomic selection models

For the GS proof-of-concept, a single-trait model was used with the corresponding ***A-*** or ***G-*** matrix for the conventional ABLUP and GBLUP models and 11 phenotypic traits evaluated. For the ABLUP model, the additive relationship matrix ***(A)*** was calculated using the getA function in pedigreemm R software (Vazquez et al., 2010). For the GBLUP model the genetic relationship matrix ***(G)*** was calculated using the function A. mat in the rrBLUP program (Endelman, 2011). ABLUP and GBLUP models were applied by kin.blup function (rrBLUP R package), thus solves mixed models of the form:

***y*** = ***Xβ*** + ***Z*** ***g*** + ***ε***

Where ***β*** is a vector of fixed effects, ***g*** is a vector of random genotypic values with covariance ***G*** = ***Var*** (***g***), and the residuals follow ***Var*** (***ε***) = ***R***_***i***_ ***σ*** ^***2***^_***e***_, with ***R***_***i***_ ***= 1*** by default. For all models and phenotypic traits, the number of trees with genotypic data was the same (280 trees). However, the number of individuals with both phenotypic and genotypic data varied by trait (Supplementary Table S2). For ABLUP only the genotyped individuals in the trial were predicted to make the results comparable to those obtained with GBLUP.

#### 2.6.2 Validation of the model

A leave-one-out (LOO) cross-validation strategy was performed for all traits, where in each case the entire population except one individual was used as the TP and the phenotype of the excluded individual was predicted. Pearson’s correlations (stats R package, cor function, R Core Team, 2023) between the phenotypic records corrected for environmental effects and the predicted values were used to obtain the predictive ability (PA) of each model. The significance of Pearson’s correlation was determined using a two-tailed *t*-test with an alpha level of 0.05.

#### 2.6.3 Heritability

For the estimation of variance components and heritability, ABLUP results from the kin.blup function of the rrBLUP package were used (Endelman, 2011).

## 3 Results

### 3.1 Genotyping data of *E. dunnii* breeding population

#### 3.1.1 SNP ddRADseq dataset

Sequencing of all 13 library pools on the NextSeq instrument yielded 383.5 million PE (57.6 Gb) passing filter reads (mean quality greater than 30 Phred index). The average number of PE reads per pool of 24 samples was 24,224,266.5.

After all sequence quality filters, an average of 1,009,344.5 PE reads per sample was finally obtained. However, there was a large variation in the number of PE reads between samples (112,342 to 3,116,508). This variation is expected and mainly due to the variation in the number of reads within sample pools (Aguirre et al., 2019).

A mean of ∼80% of reads per individual mapped to the *E. grandis* v2.0 reference genome and 530,885 SNPs at 195,010 loci (75bp each locus) were found in the SNP calling analysis. After applying the first loci and SNPs quality filters with Stacks software, a raw ddRADseq dataset was obtained, with 42,058 SNPs in 16,123 polymorphic loci. Each locus was defined by a depth of at least 6 reads, a likelihood greater than −20, a MAF greater than 0.01 and the presences in at least 50% of the 308 individuals.

#### 3.1.2 SNP EUChip60K dataset

Following the standard genotyping and quality control procedures (Illumina, 2010), all 308 individuals were successfully genotyped.

### 3.2 Genomic datasets quality filter and imputation

The overall proportions of missing data for the EUChip60K (64,639 SNPs) and ddRADseq (42,058 SNPs) datasets were 0.11 and 0.34, respectively. The proportion per individual ranged from 0.13 to 0.87 with a mean of 0.34 ± 0.15 for ddRADseq and between 0.10 and 0.14 with a mean of 0.11 ± 0.006 for the EUChip60K data. Twenty-eight individuals were eliminated from both datasets: 18 of them showed a high proportion of missing data (60%) in ddRADseq dataset; 10 of them presented heterozygosity values greater than 0.4 (mean of 308 individuals: 0.29 ± 0.04) and a closer than expected genetic relationship with another individual (two of them in both datasets and eight only in the EUChip60K dataset).

SNPs with more than 20% of missing data and MAF below 0.01 were filtered out. The final datasets were composed of 280 individuals with 8,170 ddRADseq SNPs and a total of 13% missing data and the same 280 individuals 19,045 EUChip60K with SNPs and a total of 3% missing data. The Mantel test correlation between the genetic distance matrices obtained with the two datasets was found to be r = 0.69 (Significance 0.001). LinkImpute, the software/algorithm used to impute, estimates accuracy before processing by sub-sampling the existing data, removing and imputing (Money et al., 2015). Imputation accuracies of 0.89 and 0.84 were achieved for the ddRADseq (8,170 SNPs) and EUChip60K (19,045 SNPs) dataset.

Finally, both datasets were joined, creating the combined dataset. Since the imputation could modify SNPs allele frequencies, the three datasets were again filtered by MAF, giving a total of 8,011, 19,008 and 27,019 SNPs for ddRADseq, EUChip60K, and the combined dataset, respectively.

### 3.3 Population genetics analyses

#### 3.3.1 SNPs distribution and MAF in the three datasets

**FIGURE 1:**
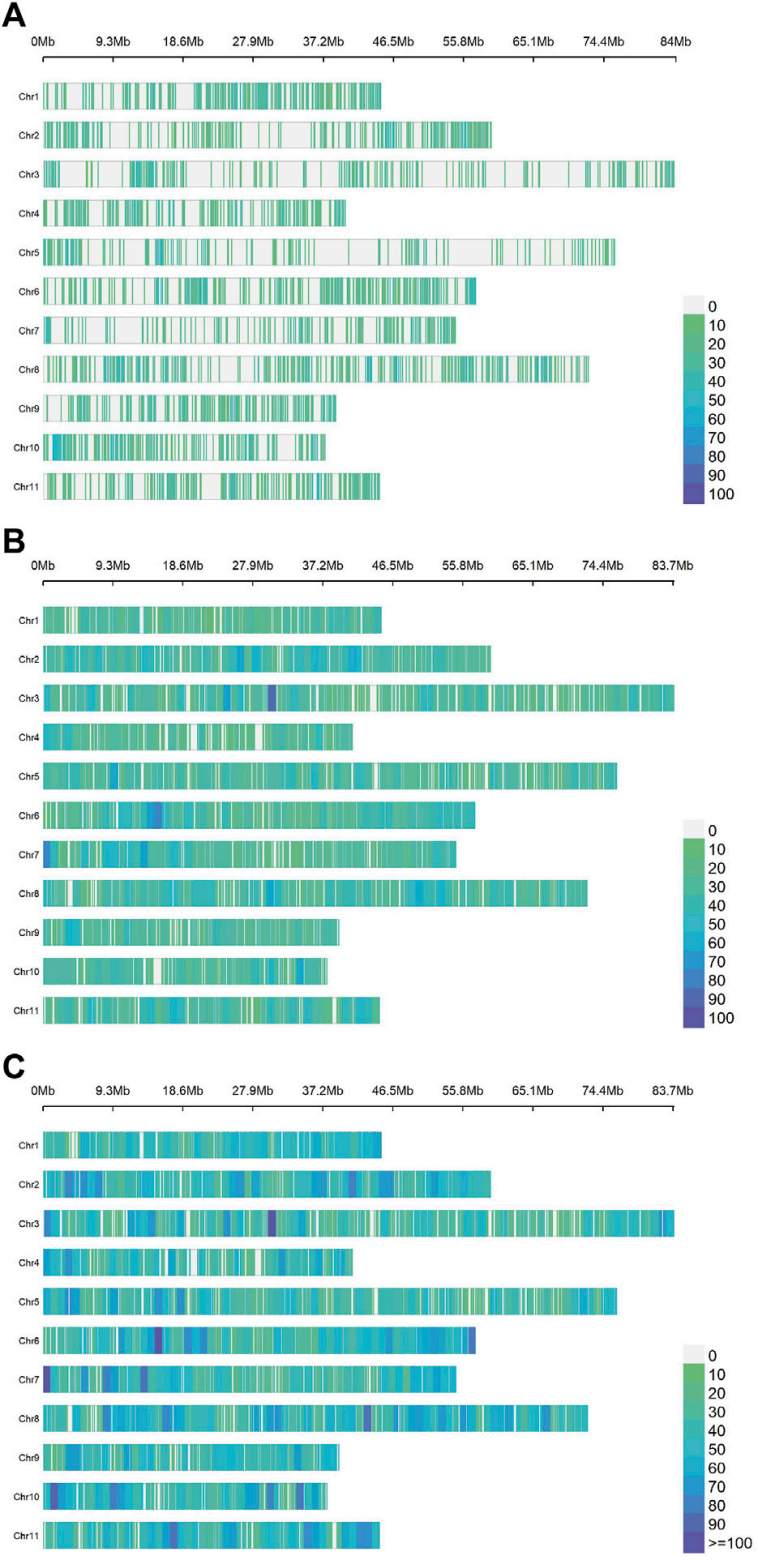
*Density and distribution of the SNPs per chromosome for each dataset of the Eucalyptus dunnii population.* The chromosomes are plotted from 1 (top) to 11 (bottom) on the ordinate axis and their sizes are given in Mb on the abscissa axis. The colour scale varies from zero SNPs (green) to more than 100 SNPs per 1 Mb (deep blue). From top to bottom: **(A)** ddRADseq (7,831 SNPs); **(B)**. EUChip60K (18,678 SNPs); **(C)**. ddRADseq + EUChip60K (26,509 SNPs).

The average number of SNPs per chromosome was 712, 1,698 and 2,410 for ddRADseq, EUChip60K and the combined dataset respectively, distributed along the 11 chromosomes (Figure 1).

Examining the mean distances between contiguous SNPs (Supplementary Table S3) and, given the lower number of markers, the ddRADseq dataset shows the largest distances (75,831 ± 20,108 bp). The EUChip60K data showed on average less than half that distance (33,198.04 ± 2,217.10 bp). The combined datasets showed the lowest inter-SNPs distance (average: 23,009.75 bp ± 2,217.10 bp). In Figure 1, the ddRADseq dataset reflected a more clustered pattern of SNP distribution than the EUChip60K dataset, again consistent with the higher mean distances between SNPs and its higher standard deviation.

In relation to the sum of the extreme SNP distance for all chromosomes (Total), it is observed that, with the combined dataset (coverage of 611.09 Mb), this value closely approximates the sizes of the *E. grandis* genome (640 Mb). It is important to note that these distances are relative, since they were estimated based on the *E. grandis* reference genome (Myburg et al., 2014) (Figure 1) which has a larger size compared to the *E. dunnii* genome (∼530 Mb, Grattapaglia & Bradshaw, 1994). These results suggest that both genotyping methods cover the whole genome, as shown in the SNP density and distribution scheme per chromosome (Figure 1).

**FIGURE 2:**
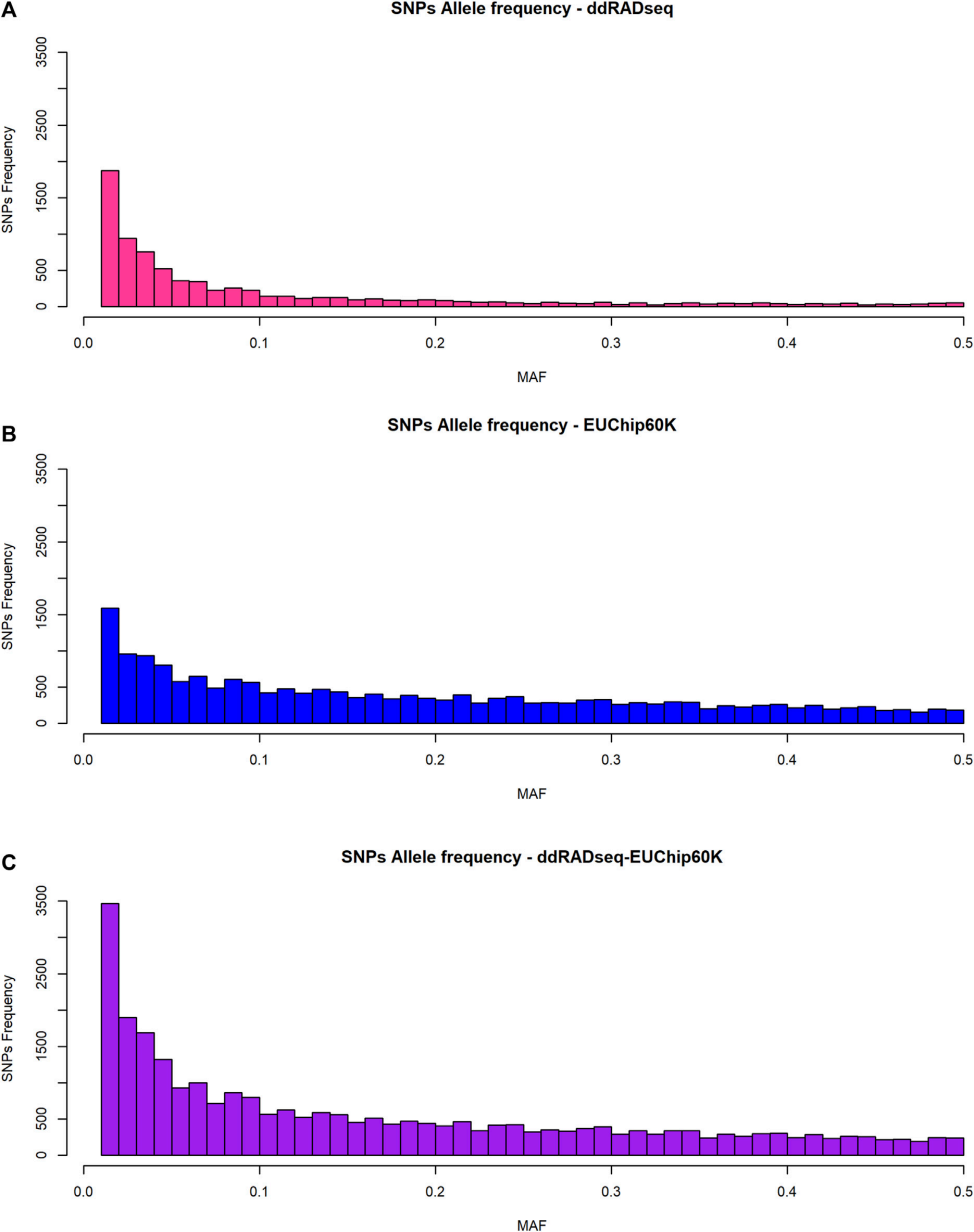
*Allele frequency distribution of SNPs.* **(A)** ddRADseq; **(B)** EUChip60K; **(C)** ddRADseq + EUChip60K.

The allele frequency spectrum of SNP data generated by the two genotyping systems showed a striking difference (Figure 2). ddRADseq showed a strong bias towards low-frequency alleles, with 68% of SNPs (5,469 of 8,011 SNPs) having a MAF <0.1, consistent with the fact that the vast majority of variants will be rare. The EUChip60K data showed the expected MAF distribution based on the preselection made during chip development aimed to enrich SNPs with higher frequency, reflecting the coverage bias in fixed-content SNP chip data (37% of the SNPs with MAF <0.1, 7,070 of 19,008 SNPs). The combined datasets provided a MAF distribution that should be of interest for the GS approach (46% of the SNPs with MAF <0.1, 12539 of 27,019 SNPs).

#### 3.3.2 Linkage disequilibrium estimation

**FIGURE 3:**
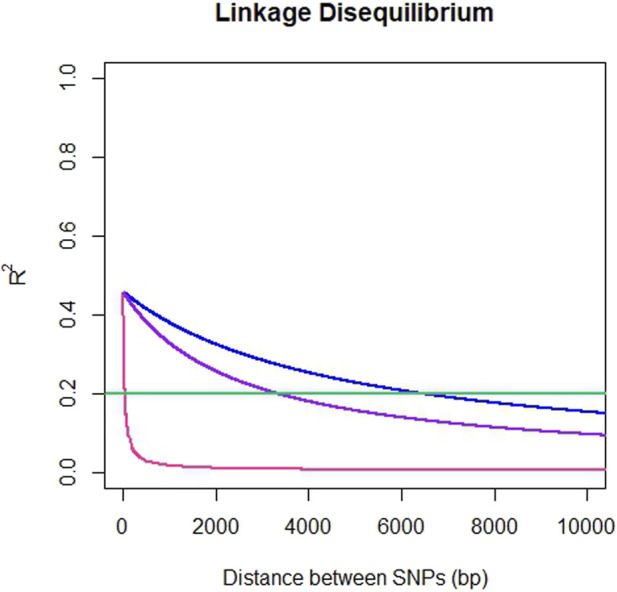
*Pattern of linkage disequilibrium decay in E. dunnii*. Pairwise SNP distances up to 10 Kbp *versus r* ^2^ for ddRADseq (pink), EUChip60K (blue) and ddRADseq + EUChip60K (purple). Abline at 0.2 *r* ^2^ in green.

The average genome-wide LD for SNP pairs (*r* ^2^) for ddRADseq, EUChip60K and ddRADseq + EUChip60K were 0.025, 0.032 and 0.033, respectively. LD was observed to fall below the 0.2 *r* ^2^ threshold at a distance of 37 bp for the ddRADseq dataset, 6,387 bp (6.4 Kbp) for EUChip60K and 3,298 bp (3.3 Kbp) for the combined dataset (Figure 3).

#### 3.3.3 Population genetic structure and diversity parameters estimation

To ensure more reliable estimates of population genetic structure parameters, SNP pairs displaying an *r* ^2^ value greater than 0.2 were pruned. The LD-pruned datasets consisted of 4,848 SNPs for ddRADseq, 13,385 SNPs for EUChip60K, and 17,611 SNPs for the combined set.

**FIGURE 4:**
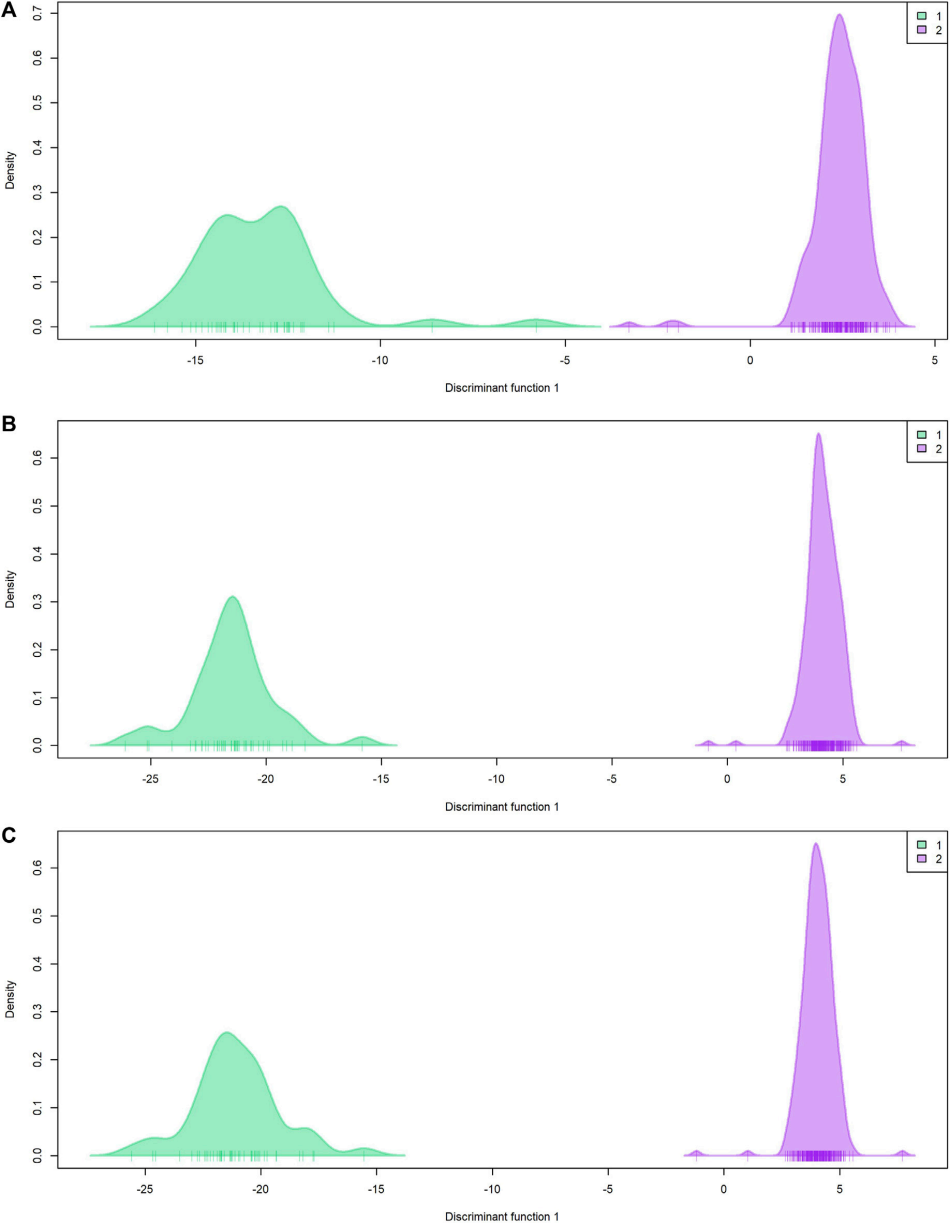
*Population genetic structure by DAPC*. Plot of density by discriminant function 1. **(A)** ddRADseq; **(B)** EUChip60K; **(C)** ddRADseq + EUChip60K. Group 1 (green) composed by 43 **(A)** or 45 **(B, C)** individuals; group 2 (violet) composed by 239 **(A)** or 237 **(B, C)** individuals.

Population genetic structure analysis with the DAPC method identified two genetic groups based on the lowest BIC value (Figure 4; Supplementary Figures 1A–C). Only two individuals differed in group assignment when datasets were compared. These two individuals were assigned to group one by EUChip60K and combined datasets, but belonged to group two when the ddRADseq dataset was applied. Group one was composed of 43 (ddRADseq) or 45 (EUChip60k and combined data-set) of the 52 individuals coming from local provenance seeds (Australian origin: Moleton, NSW; Supplementary Table S1). The group two consisted of the remaining 239 (ddRADseq) or 237 (EUChip60k and combined data-set) individuals, depending on the dataset considered (Supplementary Figures 1D–F). However, the F_ST_ estimates between these two genetic groups were low, irrespective of the SNP dataset used (F_ST_ = 0.0148, *p*-value <0.05 for ddRADseq; F_ST_ = 0.0155, *p*-value <0.05 for EUChip60K, and F_ST_ = 0.0148, *p*-value <0.05 for the combined dataset). Population genetic statistics were estimated with each SNP dataset (Table 1). Higher diversity was estimated with the SNP chip data, consistent with the higher MAF observed for the SNPs sampled. No significant difference was seen in diversity measures between the two groups found in the structure analysis.

**Table 1:** Population genetic diversity parameters estimated with the three genotyping datasets. Total pop.: parameters calculated for whole population; Group 1 and Group 2: parameters calculated for each genetic group defined by DAPC analysis; p: average major allele frequency; q: average minor allele frequency; He: expected heterozygosity; Ho: observed heterozygosity; PIC: polymorphic information content.

	ddRADseq			EUChip60K			ddRADseq+EUChip60K		
	Total pop.	Group 1	Group 2	Total pop.	Group 1	Group 2	Total pop.	Group 1	Group 2
p	0.89	0.89	0.89	0.80	0.80	0.80	0.82	0.82	0.82
q	0.11	0.11	0.11	0.20	0.20	0.20	0.18	0.18	0.18
He	0.17	0.14	0.17	0.28	0.25	0.28	0.25	0.22	0.25
Ho	0.15	0.13	0.15	0.29	0.27	0.29	0.26	0.24	0.26
PIC	0.14	0.12	0.14	0.23	0.20	0.23	0.21	0.18	0.21

### 3.4 Genomic selection

**FIGURE 5:**
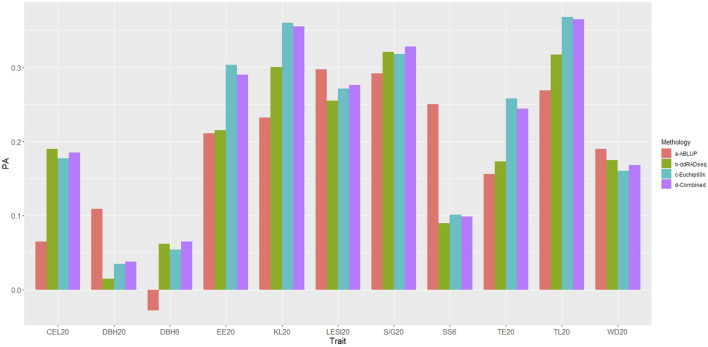
*Predictive abilities obtained with ABLUP and GBLUP (for each genomic dataset).* PA: Predictive ability. Traits under study: DBH: diameter at breast height, SS: stem shape, LESI: log end split index, EE: ethanolic extractives, TE: total extractives, KL: Klason lignin, TL: total lignin, SG: Syringyl/Guaiacyl, CEL: cellulose, WD: basic density. Methodology: a- ABLUP (red), b-ddRADseq (green), c- EUChip60K (blue), d-ddRADseq + EUChip60K (violet).

The predictive ability (PA) of GBLUP (all genomic datasets) was superior to ABLUP for six of the traits evaluated (Figure 5, Supplementary Table S4), specifically wood quality traits estimated by NIR (EE20, TE20, KL20, TL20, S/G20, CEL20). However, for the growth trait DBH6, despite GBLUP demonstrating superiority, the PA value was nearly zero and not statistically significant (*p* > 0.05). In contrast, ABLUP presented higher PA values for SS6, DBH20, LESI20 and WD20.

When comparing the GBLUP PA obtained with the ddRADseq and EUChip60K SNP datasets, the latter yielded higher and significant PA values for six traits (SS6, LESI20, EE20, TE20, KL20 and TL20), while ddRADseq dataset yielded higher PA values for three traits (S/G20, CEL20 and WD20). For both DBH traits PA values were close to zero and not significant (*p* > 0.05) with the two datasets. For six of the 11 traits PAs obtained with the EUChip60K data were higher than those obtained with ddRADseq data and for some traits such EE20, KL20, TE20 and TL20 the differences were substantial.

Contrasting the results of the combined dataset with each independent dataset, it is observed that for three traits (DBH20, LESI20 and S/G20), the former showed slightly higher PA values, although overall the PA values obtained with the combined datasets mostly close to the highest PA value obtained with one of the independent datasets.

The mean squared errors (MSEs) were similar between ABLUP and GBLUP, with the three datasets, EUChip60K and the combined dataset showing slightly lower average MSEs (ABLUP: 0.904, ddRADseq: 0.898, EUChip60K: 0.885 and ddRADseq + EUChip60K: 0.885).

Heritabilities estimated with the ABLUP model varied between 0.251 for CEL20 and 0.834 for LESI20 (Supplementary Table S4). Four traits showed high (>0.5) h^2^ values (LESI20, EE20, KL20 and TL20) while the remaining traits had moderate values (0.15 < h^2^ < 0.50). Pearson’s correlations between h^2^ and PA were 0.819, 0.732, 0.805 and 0.796 for pedigree, ddRADseq, EUChip60K and ddRADseq + EUChip60K data respectively.

## 4 Discussion

The present study aimed to evaluate SNP data obtained with two alternative genotyping methods, ddRADseq and fixed-content SNP array, for estimating population genetic parameters and modeling GS for wood quality and growth traits in a breeding population of *E. dunnii,* a forest tree. This *Eucalyptus* species is important in the context of climate change, due to its growth advantages on some environmental conditions. Having access to a high-density, low-cost, flexible, and accurate genotyping platform is essential for the successful application of GS. The number of informative markers is expected to be directly proportional to the predictive power of a GS model, by more accurately capturing relatedness between training set and selection candidates and increasing the likelihood that loci controlling the target quantitative trait will be in LD with at least one marker (Meuwissen and Goddard, 2010).

### 4.1 ddRADseq application

Due to the absence of a reference genome for *E. dunnii*, we initially evaluated SNP discovery and genotyping with both a *de novo* and a reference-based analysis to compare the results (Stacks program; Catchen et al., 2013). Both analyses can be applied to identify SNPs with high accuracy after applying stringent bioinformatics and quality filters (Aguirre et al., 2019). After implementing the first quality filters 42,058 SNPs were found in 16,123 polymorphic loci with a reference-based method. In contrast to the reference-based analysis, which only considers reads that map on the *E. grandis* genome v2.0 (80% of reads), the *de novo* analysis uses all reads for marker identification. As expected, the *de novo* analysis recovered more SNPs and loci (55,338 SNPs in 22,629 polymorphic loci). These results are encouraging to explore these *E. dunnii* SNPs that could not be detected using the *E. grandis* reference. On the other hand, *de novo* discovery requires stricter criteria and parameters when defining loci, due to the higher number of false positives obtained (Rochette and Catchen, 2017). Thus, only SNPs detected by the reference-based analysis were considered for the goal of comparing ddRADseq with EUChip60K datasets in downstream applications. Additionally, based on previous work with imputation strategies for ddRADseq data (Merino, 2018), a further filter was applied to this SNPs dataset, leaving only those with call rate >80% (Aguirre et al., 2019). These *de novo* analysis results suggest that ddRADseq has the potential to be widely applicable to forest tree species that do not have a reference genome. Nevertheless, it should be pointed out that for species without a reference genome, the sequencing data should aim for longer paired-end reads than the 75 bp long ones obtained for *E. dunnii* in this study. In addition, the number of optimal reads per sample required depends on the optimal number of loci and depth of coverage driven by the project’s goals and the genetic nature of the species under study (Andrews et al., 2016). Reliable *de novo* locus discovery and genotyping in diploids requires high coverage (10–20x or >20x; Andrews et al., 2016; Rochette and Catchen, 2017). According to the previous setup of the ddRADseq protocol for *E. dunnii* (Aguirre et al., 2019), a minimum of 700,000 reads per sample is required to achieve 10x depth coverage, although it is better to guarantee a minimum average of 1 million reads per sample to ensure good results for all samples. In our hands, as well as filtering for quality of ddRADseq loci and removing individuals with a high proportion of missing data, extreme heterozygosity and unexpected relatedness (in both datasets), this minimum average number of reads per sample was employed to acquire sturdy data for the study.

In the *Eucalyptus* genus, despite its high cost, WGS was applied in some cases (Kainer et al., 2019; Yong et al., 2021). RRS has been applied for association studies by using target re-sequencing of specific genes (Ghosh Dasgupta et al., 2021; Candotti et al., 2023). Besides, sequence capture, where *E. grandis* reference genome (Bartholomé et al., 2014; Myburg et al., 2014) was used to design probes and capture genomic regions to be sequenced, was evaluated (de Moraes et al., 2018). It is worth mentioning that, as the latter methodologies, ddRADseq has the potential and allows the discovery and detection of all kinds of DNA variation (e.g., copy number variants or CNV, microsatellites or SSRs, SNP, InDels, plastid DNA; Aguirre et al., 2019; Meger et al., 2019; Aballay et al., 2021) and the inclusion of all types of variants could improve the predictive ability in GS (Lyra et al., 2019; Della Coletta et al., 2021).

### 4.2 EUChip60K microarray application

As expected, the number of polymorphic SNPs obtained with the EUChip60K in the 308 individuals (19,011 SNPs, with Call rate >80% and MAF> 0.01) was slightly higher than the number originally reported based on genotyping only 12 individuals (17,014 SNPs; Call rate 98.8%, MAF >0.01) (Silva-Junior et al., 2015). As pointed out by Resende et al. (2017), less than 50% of the 64 thousand SNPs available in the EUChip60K are typically polymorphic in line with the multi-species nature of the EUChip60K, in which not all SNPs were designed to be informative in each single species, but rather that the chip would provide approximately 15,000 to 30,000 useful SNP in each one of almost 20 eucalypt species (Silva-Junior et al., 2015). The highest proportions of informative SNPs in the EUChip60K are generally found in species that were more represented in the sequencing data used for SNP discovery. This was the case of the work of Cappa et al. (2019), who obtained 33,398 SNPs (MAF >0.01) for 999 trees of hybrids between *E. grandis* × *E. urophylla* and *E. grandis* × *E. camaldulensis*. Conversely, for less represented species like *E. dunnii* in this work, Jurcic et al. (2021), reported 11,284 with a more rigorous MAF>0.05, and Suontama et al. (2019) reported 12,236 SNPs in 691 individuals of *E. nitens*.

### 4.3 Comparison of genotyping methodologies

#### 4.3.1 Missing data and imputation

ddRADseq was chosen to evaluate against the standard EUChip60K data because it is the REbRRS method that typically yields a higher number of reliable markers, as observed for beech and oak (Ulaszewski et al., 2021), when comparing RADseq, GBS and ddRADseq. When comparing the ddRADseq and EUChip60K datasets, the latter had a much lower proportion of total and per sample missing data. This was expected due to the EUChip60K design and DNA hybridisation-based methodology (de Moraes et al., 2018). However, in order to overcome missing data, imputation methods were applied as reported for *Picea glauca* (Gamal El-Dien et al., 2015). The LD-kNNi imputation algorithm was applied to both genomic datasets for the *E. dunnii* population. High accuracies of genotype assignment to missing data were obtained (0.8949 for ddRADseq dataset with 8,170 SNPs; and 0.8443 for EUChip60K dataset with 19,045 SNPs). Likewise, an algorithm also based on the use of nearest-neighbour genotype information, kNN-Fam, together with the Expectation Maximisation algorithm, showed the highest accuracies in imputing GBS data for GS in *P. glauca* (Gamal El-Dien et al., 2015) when compared to the mean imputation and singular value decomposition methods. On the other hand, Rutkoski et al. (2013) compared four imputation methods for application in GS and found that the random forest regression method produced superior accuracy, followed by the kNNi method, and the lowest accuracy was the mean imputation method. Furthermore, they concluded that including markers with a large proportion of missing data almost always led to higher GS accuracies after imputing, even when the order of the markers is not known (Rutkoski et al., 2013). Another study in *E. cladocalyx* also applied the LD-kNNi algorithm, implemented in 5.2 (Trait Analysis Association, Evolution and Linkage; Bradbury et al., 2007), which allowed them to impute data and apply GS in this non-model species (Ballesta et al., 2020).

#### 4.3.2 Minor allele frequencies

*Eucalyptus* species is primarily outcrossing and has wide pollen/seed dispersal. As a result, high proportions of polymorphic loci with rare alleles are observed in natural populations (Byrne, 2008). The breeding population genotyped in the present work is likely to retain a high proportion of such rare variants as it is only one generation removed from natural stands. SNPs with MAF >0.01 were retained for the downstream analyses since 4,011 of the 8,011 SNPs in the ddRADseq dataset had a frequency <0.05. SNPs with a MAF >0.01 were also used in GS in *Eucalyptus* (Cappa et al., 2019; Suontama et al., 2019). The inclusion of rare variants has the potential to contribute to the accuracy of GS prediction models, although the overall contribution of rare SNPs to the variance of quantitative traits in breeding populations has been questioned (Liu et al., 2015), and some reports suggest that low MAF SNPs, do not influence genomic predictions (Zhu et al., 2017; Zhang et al., 2019; Trujano-Chavez et al., 2021).

The multispecies strategy used for the development of EUChip60K somewhat mitigated the ascertainment bias towards more common SNPs (Silva-Junior et al., 2015). However, when comparing the MAF distributions obtained in the datasets of the two genotyping methods, EUChip60K SNPs showed a higher average allele frequency and a lower proportion of rarer SNPs than ddRADseq in this *E. dunnii* population. This corroborates the expectation that the EUChip60K targets more common polymorphisms in the population than ddRADseq. This same trend was observed in other studies comparing RRS and SNP arrays for the same sample set. Negro et al. (2019) working with maize observed that array data showed a uniform MAF distribution, while GBS data presented an excess of rare alleles with an “L” shaped MAF distribution. The authors justify these differences because maize microarrays (50K and 600K) were developed based on sequencing 27 and 30 lines respectively, while SNPs from GBS data were detected in 247 lines, allowing for a greater discovery of rare alleles. Otherwise, in *Eucalyptus*, as expected, the proportion of rare variants was significantly higher with the sequence capture method than with the EUChip60K (de Moraes et al., 2018).

#### 4.3.3 SNP density

With respect to the distribution of SNPs along the genome, it was observed that SNPs obtained from ddRADseq analysis showed a more clustered and less homogeneous pattern than those obtained with the chip data. This is consistent with the expectations based on the design of the EUchip60K. To develop it, 240 tree genomes from 12 species were sequenced at a depth of 3.5× each, resulting in a total of 46,997,586 raw SNP variants. The SNPs were filtered using multivariable metrics, retaining only variant SNPs of high quality that displayed polymorphism in the largest number of species. This resulted in an array containing 60,904 SNPs, with a homogeneous genome-wide coverage of 96% (1 SNP per 12–20 kb) as reported by Silva-Junior et al., in 2015. Similar pattern was seen when EUChip60K data was compared to sequence capture data (de Moraes et al., 2018) and also in maize, where SNPs from GBS showed higher SNP density in telomeric regions, while the 50K microarray data showed a more homogeneous distribution, and the 600K microarray showed a higher density of markers in pericentromeric regions (Negro et al., 2019). These results demonstrate the benefit of pre-selecting polymorphic loci when developing a SNP array, despite the inherent limitation of variable levels of ascertainment bias. Similarly, in *E. dunnii* no SNPs were found common to both genotyping methods, which is consistent with a study in *E. globulus* (Durán et al., 2018), where it was observed that of the 2,597 SNPs obtained with the GBS method, only 24 SNPs were common to the 13,669 polymorphic SNPs presented by EUChip60K.

#### 4.3.4 Linkage disequilibrium

Pairwise estimates of LD (*r* ^2^) between all SNPs (MAF ≥0.01) and all chromosomes were independently estimated for the three datasets. A rapid LD decay in *Eucalyptus* genus was reported in several studies, presenting values of LD that dropped below 0.2 between 5.7 Kbp to 637.7 Kbp (Silva-Junior and Grattapaglia, 2015; Muller et al., 2019; Estopa et al., 2023). *E. dunnii* showed a rapid LD decay between this range (EUChip60K dataset).

The genome-wide LD decay to an *r* ^2^ below 0.2 was significantly faster for ddRADseq (37 bp) compared to EUChip60K (6.4 Kbp). This difference can be explained by the small distances between SNPs within the same locus in the ddRADseq dataset, which were generated for 75 bp of the Illumina read length (average of 2.6 SNPs every 75bp genomic region or locus).

Similar results were obtained when comparing sequence capture with EUChip60K. The same trend was observed by de Moraes et al. (2018) for two MAF thresholds (0.05 and 0.1), falling below the 0.2 *r* ^2^ value at lower distances (50-100Kbp) for the sequence-capture SNP dataset than with the SNP array (250 Kbp) for a MAF of 0.05. They explain that these LD decay differences between datasets could be due to the intensive pre-selection step for the SNPs included in the SNP array (1 SNP every 12–20 Kbp, Silva-Junior et al., 2015) likely resulting in ascertainment bias.

#### 4.3.5 Genetic diversity

The maintenance of genetic diversity is key to the viability of a population, particularly in a breeding program where long-term sustainable genetic gain with GS should be in balance with genetic diversity (Grattapaglia, 2022). According to the MAF distribution, for *E. dunnii* a lower average heterozygosity (He) was observed with ddRADseq (0.17) than with the microarray (0.28). This difference is consistent with the observed allele frequency spectrum of the two datasets where the SNPs genotyped with the EUChip60k have a higher average allele frequency which will result in higher heterozygosity. Negro et al. (2019) observed the same trend in maize, where He for GBS (Elshire et al., 2011) was 0.27 and for the 50K and 600K microarrays was 0.35 and 0.34, respectively. The observed heterozygosity for *E. dunnii* (Ho: 0.29) with the EUChip60K dataset was similar to that found in *E. cladocalyx* with the same SNP-chip (Ho: 0.22; Ballesta et al., 2020). As expected it was lower than the estimate for an *E. dunnii* seed orchard using nine multiallelic SSR markers (Ho: 0.66; Zelener et al., 2005).

#### 4.3.6 Genetic structure

The population genetic structure detected with the two datasets was similar despite the differences in allele frequency distributions, and only two individuals differed in the genetic groups assignment. Such correspondence between the population structure obtained with the GBS and SNP-chip datasets was also observed by Negro et al. (2019) in maize and by Elbasyoni et al. (2018) in winter wheat. Such population genetic structure detected by DAPC analysis in *E. dunnii* population refers to two groups with little but significant genetic differentiation between them. This is likely due to the genetic composition of the smallest group (trees selected for growth and stem straightness in a local commercial plantation with a narrow genetic base) and the composition of the largest genetic group, which has many families from different geographical sources of seeds, resulting in dissimilar allele frequencies (Alqudah et al., 2019). However, the population genetic structure detected was very low (F_ST_ = 0.0148) although significant. As suggested by Yu et al. (2006), kinship relationships are able to capture the underlying genetic structure except in cases where there is an obvious regional difference (Cappa et al., 2013). In the case of the *E. dunnii* population under study, it is derived from seeds from a very narrow geographic region in Australia, corresponding to the distribution of the species, suggesting high gene flow and therefore little genetic differentiation.

#### 4.3.7 Genomic selection

This study compared the performance of two genomic datasets and their combination, in building a genomic selection model for a breeding population of *E. dunnii.* They were contrasted with the traditional approach using pedigree information (ABLUP). The evaluation was based on their predictive ability for 11 growth and wood quality traits. When considering the present results, it must be taken into account that the size of the population studied is rather small, a factor that affects the accuracy of the prediction (Grattapaglia, 2022).

Several studies have also applied GS in *Eucalyptus* using EUChip60K data (Müller and Neves, 2017; Durán et al., 2018; Cappa et al., 2019; Suontama et al., 2019; Jurcic et al., 2021; Duarte et al., 2023), but none of them used the ddRADseq genotyping method. This present work is the first to compare ddRADseq and SNP array data for the application of GS in forest trees and the first to apply GBLUP using ddRADseq in *E. dunnii*.

Genomic approaches are expected to perform better than pedigree-based approaches because they use more accurate kinship information (Cappa et al., 2019). Our results showed that the GBLUP (with any of the three data sets) outperformed the ABLUP for six out of 11 traits. However, the ABLUP approach performed better than GBLUP for four traits, two of which were growth traits. This may be due to an overestimation of additive variation by the ABLUP approach, which cannot disentangle the non-additive variation (Muñoz and Sanchez, 2014; Gamal El-Dien et al., 2016; Cappa et al., 2019).

Previous studies have used the EUChip60K platform and applied GBLUP and five other Bayesian GS methods to predict traits in different *Eucalyptus* populations. For instance, Müller and Neves (2017) showed predictive abilities for DBH of 0.16 and 0.44 for populations of *E. benthamii* (n = 505) and *E. pellita* (n = 732), respectively. In contrast, our study found lower and no significant PA values for DBH at 6 and 20 years in the Ubajay population of *E. dunnii* (PA with chip: 0.054 and 0.035 DBH6 and DBH20, respectively, with GBLUP). These results suggest that the *E. dunnii* population has lower additive genetic variation for this trait. Another study by Durán et al. (2018) applied GBLUP and three other Bayesian GS models to predict stem volume and wood density traits in a clonal population of *E. globulus* using the EUChip60K microarray. This population had a similar size (310 trees) to the *E. dunnii* population (280 trees). The study found a higher PA value, using GBLUP, for wood density (0.63) in *E. globulus* compared to *E. dunnii* (PA WD20: 0.160 with chip data). The difference in accuracy between the two populations might be due to *E. globulus* having closer kinship relationships and involving a smaller number of families (40 full-sib families and 13 half-sib families, produced by crossing 23 parents). This corroborates the well documented fact in a number of studies that effective population size and relationship are the main drivers of genomic prediction (Grattapaglia, 2022; Isik, 2022).

In *E. benthamii,* Estopa et al. (2023) compared different genomic prediction models with ABLUP in a population of 780 individuals from 77 families genotyped with the EUChip60K and phenotyped for five traits, including wood density, extractives content, and lignin content. They found that the PAs for ABLUP were lower than for GBLUP for all five traits, which is consistent with the results of the present work. For lignin content, the PA values were 0.23 for ABLUP and 0.34 for GBLUP, which are similar to the results of the present work (0.269 for ABLUP and 0.368 for GBLUP). For extractive content, the PA values were 0.16 for ABLUP and 0.18 for GBLUP. In comparison to *E. dunnii*, ABLUP was similar (0.156) and GBLUP was lower (0.258). For wood density, the PA values were 0.27 for ABLUP and 0.43 for GBLUP, which were higher than in the present work (ABLUP: 0.190 and GBLUP: 0.160).

Genomic selection in *E. dunnii* using EUChip60K data has only been applied in a few studies. In a preliminary study by Naidoo et al. (2018), GBLUP was applied to an *E. dunnii* population in South Africa. The study analyzed 9,102 SNP markers in 840 offspring from 89 half-sib families and applied GBLUP to predict five phenotypic traits. The results showed PA values of 0.38 for diameter at breast height and 0.51 for wood density. However, much lower values were found in the present study, which could be due to different environments, different origins, and a small number of genotyped individuals. Jones et al. (2019) investigated whether combining data from different trails could improve the accuracy of the GS model in *E. dunnii.* The study found that accuracy for diameter at breast height increased by 86% and tree height by 290% (from 0.18 to 0.72). Jurcic et al. (2021) applied GS in *E. dunnii* using multiple-trait multiple-site single-step GBLUP (ssGBLUP models) for DBH6 and SS6. The h^2^ of DBH6 obtained by ABLUP was 0.262 (s.d.: 0.039), which is similar to the present work results (0.242), and the PA was 0.324, but near zero in the present work. For SS6, the h^2^ was 0.19 and the PA was 0.350, while in the present work, the h^2^ was 0.413 and PA 0.25 for the same trait. However, Jurcic et al. (2021) applied a model using both genomic and pedigree information, based on a different number of individuals in the population (1,520 trees), and two additional trials, such that the PA and h^2^ values are not directly comparable.

In general, it can be concluded that models including genomic data are promising for the application in breeding programs, in particular in *E. dunnii*, as they show higher PA for most of the traits, compared to ABLUP. These models can be used to generate a ranking of *E. dunnii* individuals based on the priorities of the breeding program, such as selecting individuals with high wood quality and higher growth. The choice of genotyping platform is a key element that can affect the performance of GS (Elbasyoni et al., 2018). Sequencing-based genotyping methods in principle provide a large number of molecular markers, but often have a high proportion of missing data requiring rigorous filtering that often result in an operationally lower number of SNPs when compared to chip-based data. SNP arrays, on the other hand, provide large number of markers with very little missing data, but due to their fixed content may suffer from ascertainment bias in allele frequencies and do not allow the discovery of population-specific variants (Albrechtsen et al., 2010; Li and Kimmel, 2013; Bajgain et al., 2016). Elbasyoni et al. (2018) compared the performance of these two genotyping platforms for GS in 299 lines of winter hard wheat (*Triticum aestivum L.*), one of the few studies that compared the performance of these genotyping methods for GS in crops. They observed that GBS, imputing 10% (10,775 SNPs) and 50% (39,674 SNPs) of missing data, showed similar or even higher genomic prediction accuracy than the microarray data (19,515 SNPs) for all agronomic traits, depending on the percentage of missing data imputed from the starting GBS. In contrast, for *E. dunnii*, although ddRADseq showed slightly higher PA for some traits, the EUChip60K data provided higher PA values. This suggests that the performance of different genotyping platforms can vary depending on the species and population being studied. In the present work, it was observed that EUChip60K provided higher PA for most of the traits compared to ddRADseq. A similar trend was observed in the study by de Moraes et al. (2018), where the performance of sequence capture and EUChip60K was compared for GS. The study found that the microarray method showed higher PA for most traits.

There are various factors that affect the accuracy of prediction models, and one of them is the genotyping density (Grattapaglia, 2022). The EUChip60K dataset was found to be the most effective in predicting most of the traits evaluated in this study. This could be related to its higher marker density, which can better explain phenotypic variation compared to ddRADseq for most of the traits evaluated. Nevertheless, for two traits, the ddRADseq + EUChip60K dataset showed a higher PA, indicating that the number of markers is not the only factor that influences the PA. Kinship also plays an important role and studies on forest trees demonstrate that moderate genotyping densities of around 10,000 to 15,000 data points are sufficient for reasonable predictive power (Grattapaglia, 2022). A similar observation was made by de Moraes et al. (2018), where the combined datasets of sequence capture and EUChip60K did not improve the accuracy of the model.

Growth traits, such as diameter at breast height, are likely to be related to fitness and are controlled by a large number of genes (Falconer and Mackay, 1996) with a large interaction with the environment therefore expressing a low heritability (Nunes et al., 2016). Diameter at breast height DBH showed low to moderate h^2^, consistent with reports in other eucalypts with values between 0.11 and 0.41 (Gallo et al., 2018; Cappa et al., 2019; Marco de Lima et al., 2019; Jurcic et al., 2021). Chemical traits, on the other hand, are often related to specific biosynthesis pathway, likely controlled by fewer loci, less influenced by the environment with higher heritability (Gion et al., 2011). The heritability estimated from the pedigrees for growth and wood quality traits were moderate to high, with LESI20 and TL20 showing higher values than most wood quality traits estimated from NIR analysis or growth, in agreement with other evaluations in *Eucalyptus* (Resende et al., 2017; Tan et al., 2017; Cappa et al., 2019; Marco de Lima et al., 2019), and *E. dunnii* (Jurcic et al., 2021).

Heritability for wood density, S/G ratio and extractive content in the *E. dunnii* population were generally estimated in the same range as in previous studies of other *Eucalyptus* species (Stackpole et al., 2011; Makouanzi et al., 2017; Resende et al., 2017; Tan et al., 2017; Varghese et al., 2017; Gallo et al., 2018; Marco de Lima et al., 2019; Cappa et al., 2019; Suontama et al., 2019; Paludeto etal., 2021). For *E. dunnii*, Gallo et al. (2018) observed high broad-sense individual heritability estimates (0.64) for Klason lignin. NIR estimates of KL yielded moderate to high narrow heritability values in half-sib progeny of *E. camaldulensis* (0.21), *E. globulus* (0.27) and *E. urophylla* (0.76) (Stackpole et al., 2011; Hein et al., 2012; Varghese et al., 2017). Similar results were found for *E. dunnii* in this paper, where narrow h^2^ showed high values (KL: 0.669 and TL: 0.726), which are also in the range of the literature (de Moraes et al., 2018; Cappa et al., 2019; Marco de Lima et al., 2019). These results suggest that this trait has a high level of genetic control with a better possibility of obtaining significant genetic gains. This is also evidenced by the highest PAs obtained by genomic selection in this study. A positive correlation between heritability and predictive ability was observed in *E. dunnii* regardless of the genotyping data used. Furthermore, this correlation has already been demonstrated by Grattapaglia and Resende (2010) by means of simulations. This trend has also been observed for *Eucalyptus* (de Moraes et al., 2018), pine (Resende et al., 2012), animals (Hayes et al., 2014) and crops (Poland et al., 2012; Crossa et al., 2013).

In summary, when comparing the ddRADseq and EUChip60K methodologies, we observed differences in the percentage of missing data, genome-wide marker coverage, minor allele frequency ratios and in the estimation of genetic diversity parameters. Furthermore, no major differences were observed in the estimation of genetic structure and linkage disequilibrium.

Regarding their performance in GS, the inclusion of any of the three genomic data sets in the prediction models increases the predictive ability of the estimates compared to traditional methods. This trend was observed for most of the traits evaluated that showed significant values (six out of ten), all of them being wood quality traits. This indicates an advantage of using genomic data for selection. When comparing the ddRADseq and EUChip60K datasets, the EUChip60K yielded higher predictive abilities in most cases, although ddRADseq provided slightly higher predictions for some traits.

Both genotyping methods, ddRADseq and EUChip60K, are generally comparable for diversity analysis and genomic prediction, demonstrating the usefulness of the former provided that it undergoes rigorous SNP filtering. The results of this study provide a foundation for future whole-genome studies using ddRADseq in non-model forest species for which SNP arrays have not been developed.

## Data Availability

The data presented in the study are deposited in the European Variation Archive (EVA) in the EMBL-EBI repository, accession number PRJEB73817 (https://www.ebi.ac.uk/eva/?eva-study=PRJEB73817).

## Footnotes

1
https://www.thermofisher.com/order/catalog/product/551134; accessed on 13 December 2023

2
https://github.com/YinLiLin/R-CMplot
