# Supplementary material for: Comparison of ddRADseq and EUChip60K SNP genotyping systems for population genetics and genomic selection in Eucalyptus dunnii (Maiden)
Source: Front Genet. 2024 Mar 26;15:1361418. doi: 10.3389/fgene.2024.1361418 (PMC11008695; doi:10.3389/fgene.2024.1361418)
Supplement: Supplementary file 1 [file Table1.DOCX]

Supplementary Material

# Supplementary Figures and Tables

## Supplementary Figures


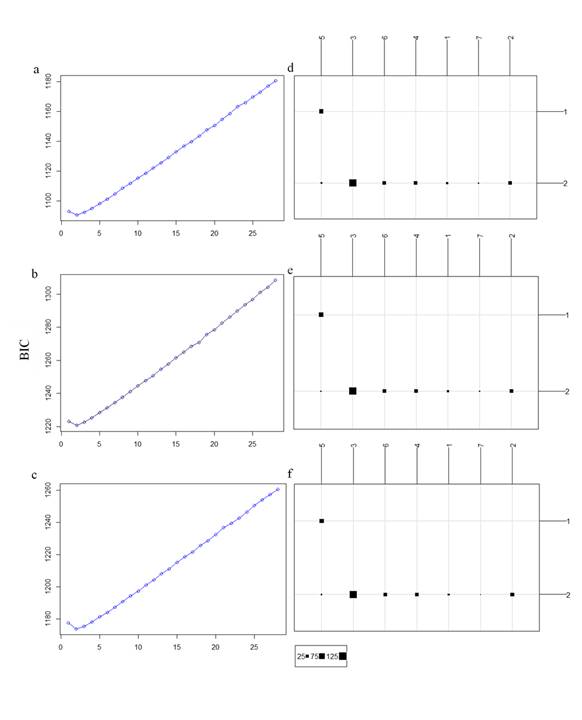


**Supplementary Figure 1.** *Population genetic structure inferred by DAPC*. References: a, b and c: Graph of BIC values versus genetic group numbers; d, e and f: Correspondence between the 7 Australian origins and 2 genetic groups inferred by DAPC for individuals from the *E. dunnii* breeding population. Each line corresponds to the analysis with ddRADseq, EUChip60K and ddRADseq+EUChip60K matrix, respectively. Vertical axis: Seed source of from New South Wales (Australia) and from local provenance plantations: 1: Acacia Creek; 2: Boomi Creek; 3: Dearth Horse Track Region; 4: Oaky Creek, NSW; 5: Local provenance; 6: South Yabra S. F.; 7: Unumgar S.F. Horizontal axis: inferred genetic groups 1 and 2.

## Supplementary Tables

| Seed source | Origin/Provenance | GeogrGeographical coordinates | Altitude (m) | N° Families |
| --- | --- | --- | --- | --- |
| BCUN | Boomi Creek, NSW | 28° 25’/152° 41’ East | 300 | 10 |
|  | Unumgar State Forest, 10 km East of Woodenbong, NSW | 28° 25’/152° 42’ East | 300 | 2 |
| DHAC | Death Horse Track Region, East of Legume, NSW | 28° 25’/152° 20’ East | 600–700 | 26 |
|  | Acacia Creek, at 25 km Northwest of Urbenville, NSW | 28° 23’/152° 20’ East | 600–750 | 4 |
| OC | Oaky Creek, NSW | 28° 36’/152° 31’ East | 520 | 9 |
| SY | South of Yabra State Forest, NSW | 28° 36’/152° 29’ East | 540 | 9 |
| SD | Local provenance: Selected trees from a commercial plantation of Oliveros, Santa Fe, Argentina (Australian origin: Moleton, NSW) | 32° 33’ 60° 51’ West (Australian origin: 30° 10’/152° 10’ East) | 27 (Australian origin: 420) | 12 |

**Supplementary Table 1.** *Seed source of E. dunnii from the Ubajay breeding population*. Seed source; origin/provenance: native populations of New South Wales (NSW) in Australia, and local provenance in Argentina; Geographical coordinates (latitude and longitude); Altitude; N° Families: number of trees harvested for each origin/provenance, corresponding to a family of half-siblings in OP. Source: López et al. (2012).

|  | Trait | Age | Code | n | Measurement unit | Mean | s.d. | Median | Min. | Max. |
| --- | --- | --- | --- | --- | --- | --- | --- | --- | --- | --- |
| 1 | **Diameter at breast height** | 6 | DBH6 | 1458 | cm | 16.33 | 3.07 | 16.30 | 6.00 | 27.00 |
| 3 | **Stem straightness** |  | SS6 | 1458 | 1 to 4 | 2.54 | 1.06 | 3.00 | 1.00 | 4.00 |
| 5 | **Diameter at breast height** | 20 | DBH20 | 318 | cm | 35.31 | 5.59 | 35.00 | 21.70 | 56.00 |
| 7 | **Log end split index** |  | LESI20 | 318 |  | 1.01 | 0.59 | 0.87 | 0.21 | 3.51 |
| 8 | **Ethanolic extractives** |  | EE20 | 269 |  | 3.29 | 1.20 | 3.10 | 1.00 | 8.80 |
| 9 | **Total extractives** |  | TE20 | 269 |  | 5.09 | 1.41 | 4.90 | 2.10 | 11.40 |
| 10 | **Klason Lignin** |  | KL20 | 269 |  | 24.99 | 0.66 | 24.90 | 23.00 | 27.00 |
| 11 | **Total Lignin** |  | TL20 | 269 |  | 28.44 | 1.04 | 28.40 | 24.80 | 31.80 |
| 12 | **Syringyl/Guayacil ratio** |  | S/G20 | 269 |  | 1.83 | 0.07 | 1.83 | 1.62 | 2.01 |
| 13 | **Total Cellulose** |  | CEL20 | 269 |  | 46.86 | 1.20 | 46.90 | 42.70 | 50.10 |
| 14 | **Wood Basic density** |  | WD20 | 269 | Kg/m^3^ | 516.52 | 67.43 | 523.80 | 313.00 | 666.90 |

**Supplementary Table 2.** *Descriptive statistics of the 11 phenotypic traits measured in the E. dunnii breeding population*. Trait; Age: age of measurement in years; code or abbreviation; n: number of individuals with data; measurement unit. The first seven traits’ data were taken from Marcó & White (2002) and López et al. (2016).

|  | ddRADseq | | | EUChip60K | | | ddRADseq+EUChip60K | | |
| --- | --- | --- | --- | --- | --- | --- | --- | --- | --- |
| Chrom. | **N° SNPs** | **Distance between extreme SNPs (Mb)** | **Average distance between contiguous SNPs (bp)** | **N° SNPs** | **Distance between extreme SNPs (Mb)** | **Average distance between contiguous SNPs (bp)** | **N° SNPs** | **Distance between extreme SNPs (Mb)** | **Average distance between contiguous SNPs (bp)** |
| 1 | 742 | 44.72 | 60355 | 1184 | 44.90 | 37921 | 1926 | 44.90 | 23312 |
| 2 | 715 | 59.17 | 82866 | 2113 | 59.39 | 28106 | 2828 | 59.39 | 21000 |
| 3 | 778 | 83.51 | 107472 | 2335 | 83.65 | 35823 | 3113 | 83.65 | 26870 |
| 4 | 615 | 40.02 | 65187 | 1050 | 41.03 | 39076 | 1665 | 41.03 | 24643 |
| 5 | 633 | 75.82 | 119976 | 2248 | 76.20 | 33898 | 2881 | 76.23 | 26458 |
| 6 | 810 | 57.26 | 70779 | 1899 | 57.34 | 30194 | 2709 | 57.37 | 21178 |
| 7 | 614 | 54.48 | 88869 | 1707 | 54.76 | 32078 | 2321 | 54.76 | 23593 |
| 8 | 1059 | 71.73 | 67799 | 2410 | 72.24 | 29976 | 3469 | 72.33 | 20849 |
| 9 | 572 | 38.30 | 67071 | 1136 | 39.18 | 34491 | 1708 | 39.18 | 22940 |
| 10 | 657 | 37.36 | 56949 | 1145 | 37.59 | 32829 | 1802 | 37.59 | 20862 |
| 11 | 636 | 43.97 | 69242 | 1451 | 44.67 | 30782 | 2087 | 44.67 | 21402 |
| Scaffolds | 180 | --- | --- | 330 | --- | --- | 510 | --- | --- |
| Avg. | 712 | 55.12 | 75831 | 1698 | 55.54 | 33198.04 | 2410 | 55.55 | 23009.75 |
| Max. | 1.059 | 83.51 | 119976 | 2410 | 83.65 | 39076.50 | 3469 | 83.65 | 26870.21 |
| Min. | 572 | 37.36 | 56949 | 1050 | 37.59 | 28106.46 | 1665 | 37.59 | 20849.01 |
| S.D. | 137 | 16.09 | 20108 | 528.65 | 15.96 | 3441.94 | 661.7 | 15.97 | 2217.10 |
| Total | 8011 | 606.62 | / | 19008 | 610.95 | / | 27019 | 611.09 | / |
| Total  WS | 7831 | --- | --- | 18678 | --- | --- | 26509 | --- | --- |

**Supplementary Table 3.** *Number of SNPs and distances between them for the three genotypic matrices.* Chrom.: Chromosomes 1 to 11 of the *E. grandis* genome, zero corresponds to the Scaffolds that could not be assembled to any chromosome in the reference; N°SNPs: Number of SNPs per chromosome; Distance between extreme SNPs (Mb): Distance between the first SNPs and the last one in each chromosome, in Mega base pairs; Average distance between contiguous SNPs in base pairs; Avg. : average of the means of all the chromosomes; Max: Maximum of the means of all the chromosomes; Min: Minimum of the means of all the chromosomes; S.D.: Standard deviation; Total: sum of all the SNPs or Mb along all the chromosomes; Total WS: Total SNPs without scaffolds.

| Trait | n | h^2^ | SD | ABLUP | | | GBLUP | | | | | |
| --- | --- | --- | --- | --- | --- | --- | --- | --- | --- | --- | --- | --- |
|  |  |  |  |  |  |  | **ddRADseq** | **ddRADseq** | **EUChip60K** | **EUChip60K** | **ddRADseq**  **+EUChip60K** | **ddRADseq**  **+EUChip60K** |
|  |  |  |  | **PA** | | **MSE** | **PA** | **MSE** | **PA** | **MSE** | **PA** | **MSE** |
| DBH6 | 280 | 0.263 | 0.002 | -0.028 | 0.948 | | 0.062 | 0.924 | 0.054 | 0.926 | **0.065** | 0.924 |
| SS6 | 280 | 0.467 | 0.002 | **0.250*** | **0.581** | | 0.09 | 0.621 | 0.101* | 0.620 | 0.099 | 0.620 |
| DBH20 | 280 | 0.291 | 0.013 | **0.109*** | **0.814** | | 0.015 | 0.830 | 0.035 | 0.829 | 0.038 | 0.828 |
| LESI20 | 280 | 0.834 | 0.015 | **0.297*** | **0.920** | | 0.255* | 0.944 | 0.271* | 0.935 | 0.276* | 0.932 |
| EE20 | 269 | 0.647 | 0.015 | 0.211* | 0.990 | | 0.215* | 0.987 | **0.303*** | 0.940 | 0.290* | 0.948 |
| TE20 | 269 | 0.496 | 0.014 | 0.156* | 0.999 | | 0.173* | 0.991 | **0.258*** | 0.954 | 0.244* | 0.961 |
| KL20 | 269 | 0.669 | 0.015 | 0.232* | 0.893 | | 0.300* | 0.858 | **0.360*** | 0.820 | 0.355* | 0.824 |
| TL20 | 269 | 0.726 | 0.012 | 0.269* | 0.887 | | 0.317* | 0.860 | **0.368*** | 0.827 | 0.365* | 0.829 |
| S/G20 | 269 | 0.549 | 0.013 | 0.292* | 0.921 | | 0.321* | 0.903 | 0.318* | 0.905 | **0.328*** | 0.898 |
| CEL20 | 269 | 0.251 | 0.011 | 0.065 | 0.975 | | **0.190*** | 0.941 | 0.177* | 0.947 | 0.185* | 0.944 |
| WD20 | 269 | 0.361 | 0.013 | **0.190*** | **1.015** | | 0.175* | 1.023 | 0.160* | 1.028 | 0.168* | 1.025 |
| DBH6 | 280 | 0.263 | 0.002 | -0.028 | 0.948 | | 0.062 | 0.924 | 0.054 | 0.926 | **0.065** | 0.924 |

**Supplementary Table 4.** *ABLUP and GBLUP predictive abilities obtained for each datasets*. Traits under study: DBH: diameter at breast height, SS: stem shape, LESI: log end split index, EE: ethanolic extractives, TE: total extractives, KL: klason lignin, TL: total lignin, SG: Syringyl/Guaiacyl, CEL: cellulose, WD: basic density. The number on the trait indicates the age of measurement. n: number of individuals with genotypic and phenotypic data. h^2^: narrow sense heritability. SD: standard deviance of heritability. PA: predictive ability. MSE: mean square error. ABLUP: values of PA and MSE obtained for the ABLUP model. GBLUP: values of PA and MSE obtained for the GBLUP model using each SNP dataset: ddRADseq, EUChip60K and ddRADseq+EUChip60K. Significant correlation values are marked with an asterisk. The highest PA values are indicated in bold and underlined are the highest values for GBLUP.
